# Formula-Driven, Size-Tunable Synthesis of PMMA Nanoparticles by Varying Surfactant Concentration

**DOI:** 10.3390/ma13081834

**Published:** 2020-04-13

**Authors:** Brian L. Kamras, Nooshin Mirzanasiri, Daniel K. Korir, Sujata Mandal, Shreya L. Hariharakumar, Robby A. Petros, Sreekar B. Marpu, Denise P. Simmons, Mohammad A. Omary

**Affiliations:** 1 Department of Chemistry, University of North Texas, Denton, TX 76203, USA; briankamras@my.unt.edu (B.L.K.); nooshin.mirzanasiri@unt.edu (N.M.); danielkorir@my.unt.edu (D.K.K.); sujatamandal@my.unt.edu (S.M.); shreyahari12@gmail.com (S.L.H.); sreekarbabu.marpu@unt.edu (S.B.M.); denise.simmons@unthsc.edu (D.P.S.); 2 Department of Mechanical and Energy Engineering, University of North Texas, Denton, TX 76203, USA; 3 Department of Chemistry and Biochemistry, Texas Woman’s University, Denton, TX 76204, USA; rpetros@twu.edu

**Keywords:** poly(methyl methacrylate) (PMMA), PMMA nanoparticles (PMMANPs), amine-functionalized block-copolymer PMMANPs (H_2_N-PMMANPs), nanoparticle size control

## Abstract

In this communication, we present a streamlined, reproducible synthetic method for the production of size-tunable poly(methyl methacrylate) (PMMA) nanoparticles (PMMANPs) and amine-functionalized block-copolymer PMMANPs (H_2_N-PMMANPs) by varying subcritical concentrations (i.e., below the concentration required to form micelles at 1 atm and 20 °C) of sodium dodecyl sulfate (SDS). We plotted the Z-average size data against SDS concentration, which revealed a second-order exponential decay function, expressed as $A_{1}e^{\left(-\frac{x}{t_{1}}\right)}+A_{2}e^{\left(-\frac{x}{t_{2}}\right)}+y_{0}$. The surfactant concentration (wt./wt.%) has been selected as independent variable x. This function is valid at least for the size range of 20 nm to 97 nm (PMMANPs) and 20 nm to 133 nm (H_2_N-PMMANPs).

## 1. Introduction

Mammalian cells, including various kinds of tumor cells, kidney, and immune system cells, show differential uptake of nanoparticles based on size and shape [1,2,3,4,5]. Nanoparticle-cell interactions are still poorly understood as a function of cell type and nanoparticle properties (e.g., size, shape, surface chemistry), and much progress needs to be made regarding these fundamental interactions. The synthesis of particles that were used in biological investigations incorporating nanomaterials is often overlooked and outsourced to companies specializing in nanoparticle synthesis, but researchers require affordable nanoparticles with customized size, surface chemistry, and surface charge. Recent studies have shown that, in addition to chemical structure, nanoparticle size affects cellular uptake [2]. Cells can experience more toxic effects from certain sizes of nanoparticles due to size-dependent nanoparticle uptake, which is a critical factor in developing cell-type specific anticancer therapies. Poly(methyl methacrylate) (PMMA) nanoparticles (PMMANPs) are an ideal nanoparticle platform for understanding the relationship between size and toxicity to various cell types because of the nanoparticles’ lack of surface functionality (e.g., functional groups, such as -OH, -NH_2_, -SH, carboxylates, and amides) and chemical non-toxicity.

PMMA is an FDA approved synthetic polymer glass that has been used for decades in dentistry, ophthalmology, and orthopedic surgery as bone cement [6]. PMMANPs are used in biomedicine as a platform for drug/gene delivery and sensing [7,8,9]. We propose that PMMANP and amine-functionalized block-copolymer PMMANP (H_2_N-PMMANP) size can be regulated while using sodium dodecyl sulfate (SDS) and center our hypothesis on previous reports in the literature that have corroborated the effect of surfactant concentration on the diameter of polymeric nanoparticles; reports from these labs reveal that, as the surfactant concentration increases, the nanoparticle diameter decreases [10,11,12]. Although surfactant-free emulsion polymerization (SFEP) has been shown to create PMMANPs in the size range of 100 nm to 1000 nm, particles that are synthesized by this method have the disadvantage of relatively high dispersities [13]. Herein, we unfold a mathematical relationship between particle size and the surfactant, SDS, concentration. To the best of our knowledge, there have been no reports of size-tunability of PMMANPs while using SDS that provide a useful mathematical relationship between particle size and surfactant concentration. In this study, we report, for the first time, a quantitative, formula-driven method to deduce PMMANP or H_2_N-PMMANP diameter as a function of SDS concentration. This facile method stands in stark contrast to more advanced microfluidics-based methods, which require costly equipment and deliver particles with a demonstrated lower variance of size [14,15]. By using the method detailed herein, researchers can precisely control in-house selected particle sizes by varying SDS concentration.

In addition to PMMANPs, we have performed the same investigation on H_2_N-PMMANPs to show that size-tunability exists for more useful block-copolymer nanoparticles, which can be covalently bound to commercial linker systems. We have analyzed the effect of varying SDS concentration on PMMANP size and H_2_N-PMMANP size by using the dynamic light scattering (DLS) technique to isolate this mathematical relationship. DLS is a powerful and noninvasive technique for measuring the size and size distribution of dispersed or dissolved particles in liquids [16]. Even though scanning electron microscopy (SEM) has been widely-used for the measurement of particle sizes and morphology, but as secondary electrons used in this technique—unlike transmission electron microscope (TEM)—it is not capable of measuring particle sizes in the nanometer range, so it is especially employed in microparticle characterization [17,18]. On the other hand, for both SEM and TEM analysis, the sample needs to be dried and coated with 5 to 10 nm of conductive materials, such as gold or platinum, which affects the hydration/dehydration, agglomeration/monodispersion, and increase/decrease of particle size due to the presence/absence of coating material, so as to preclude reliable size measurement comparisons with those that were obtained from DLS [19]. Therefore, in this study, the particle size and size distribution were measured in aqueous suspensions primarily using DLS, whereas we have only utilized SEM to show a qualitative size agreement and to shed insights on the general morphology of PMMANPs.

The Z-average diameter was tuned from 31 nm to 97 nm (PMMANP) and 34 nm to 133 nm (H_2_N-PMMANP). In both NP ranges, Z-average size follows a second-order exponential decay pattern that is dependent upon surfactant concentration. The observed dependence of size upon surfactant concentration is consistent with other reports detailing PMMANP synthesis and block-copolymer nanoparticles [13,20]. However, these groups report either a linear response of size to surfactant concentration or a single exponential decay response. The effect of surfactant concentration upon particle size might be attributable to the concentration of SDS used in our study, or the SDS concentration relative to monomer concentration in the size range investigated herein.

## 2. Materials and Methods

The synthetic method of both the functionalized and non-functionalized particles was based on batch emulsion polymerization a previously reported procedure, which we have modified to decrease the possibility of premature polymerization [10]. Namely, we have employed microwave heating rather than conventional heating to allow for a better control over power input. We have also employed the following: freeze-pump-thaw (FPT) deoxygenation using standard Schlenk techniques in place of a nitrogen gas purge, and the addition of reagents to microwave vessels in an air-free glovebox. These changes prevent accidental oxidation of potassium persulfate (KPS) by oxygen, which would cause premature polymerization.

Ultrapure water (18.2 mΩ, <2 ppb TOC) and methyl methacrylate (MMA, St. Louis, MO, USA) were deoxygenated by a triplicate FPT cycle and then added to an air-free, oxygen-free MBraun Unilab glovebox filled with a nitrogen atmosphere. Solid reagents (potassium persulfate [KPS, Sigma], SDS [Sigma], and 2-(Aminoethyl)-methacrylamide [AMA, Sigma]) were separately added to the glovebox. PMMANPs and H_2_N-PMMANPs were both heated in an Anton Paar Synthos 3000 microwave at 90 °C and 1200 W for one hour. All of the size points of PMMANPs and H_2_N-PMMANPs were synthesized in triplicate.

For the synthesis of PMMANPs, ultrapure water (29.70 mL), SDS (amount varies), MMA (0.300 mL, 2.80 mmol), and finally KPS (0.1000 g, 0.369 mmol) were loaded into teflon-lined microwave vessels inside the glovebox. The vessels were sealed, removed from the glovebox, and then microwaved.

**Figure 1:**
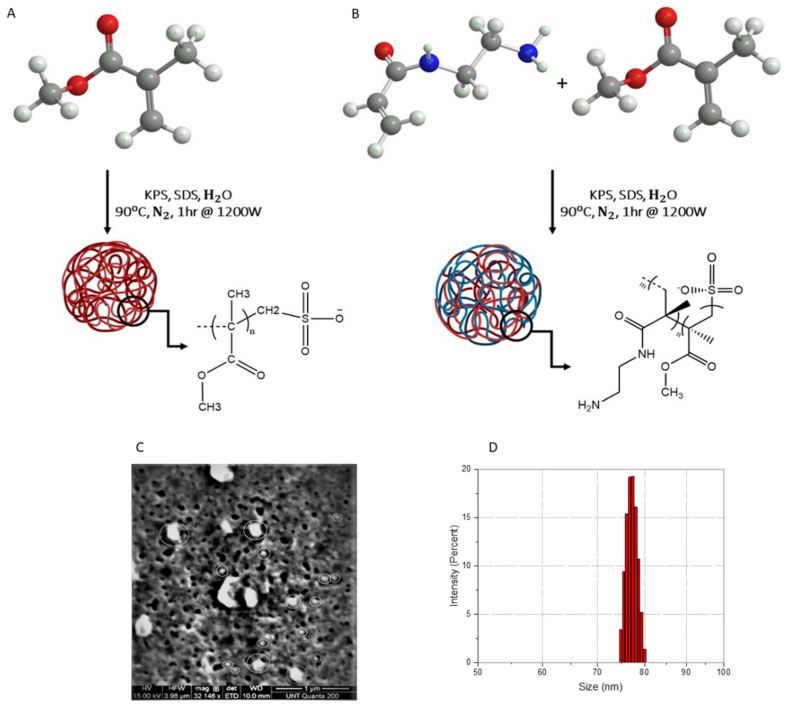
Reaction scheme illustrating the formation of size-tunable PMMANPs (**A**) and H_2_N-PMMANPs (**B**). SEM micrograph of PMMANPs produced with 0.013 wt./wt.% of sodium dodecyl sulfate (SDS) (**C**) and the size distribution by intensity obtained from DLS measurements for the same sample (**D**).

For the synthesis of H_2_N-PMMANPs, ultrapure water (29.67 mL), SDS (amount varies), MMA (0.300 mL, 2.80 mmol), AMA (0.003 g, 0.0182 mmol), and finally KPS (0.1000 g, 0.369 mmol) were loaded into teflon-lined microwave vessels inside the glovebox. The vessels were sealed, removed from the glovebox, and then microwaved. Figure 1 outlines the synthetic protocol and representative scanning electron microscopy (SEM) and dynamic light scattering (DLS) size distribution data for the particles. Figure 1A,B illustrate the schematics for the formation of size-tunable PMMA and PMMA-NH_2_ nanoparticles, respectively.

The particles were serially diluted from their initial concentrations across three orders of magnitude, and particle size was measured while using a Malvern Zetasizer (Malvern Panalytical Ltd., Malvern, WR, UK) equipped with disposable polycarbonate cuvettes. The particles were measured in triplicate, and the average of these replicates was reported as the actual value in Table 1 and Table 2. The particles were stored in the dark in a laboratory cabinet at ambient temperature (22 °C to 27 °C) after characterization. The particles were further characterized using FEI quanta 200 ESEM (Environmental Scanning Electron Microscope) (FEI Company, Hillsboro, OR, USA). The samples were loaded on aluminum grids that were covered with copper tape. On drying, a conductive layer was deposited on the samples using a Gatan 682 (Gatan, Inc., Pleasanton, CA, USA) precision etching and coating system. For higher magnification images, the samples were analyzed using FEI Technai G2 F20 S/TEM microscope (FEI Company, Hillsboro, OR, USA). However, using Formvar/carbon-coated copper grids, we observed the rupture of carbon films, resulting in the failure of analysis. The samples were not analyzed further using TEM due to accessibility and technical issues, as high-energy electron beam damaged the thin film of PMMANP on the TEM grid.

The SEM images for PMMANPs, produced with 0.013 wt./wt.% of SDS, are shown in Figure 1C. The image clearly demonstrates the spherical shape of the nanoparticles, as expected from DLS measurements (Table 1 and Table 2). Other than the particle shape, there is no additional information obtained with respect to the surface morphology of the particles at this time from SEM images, as that usually requires high-resolution transmission electron microscopy (HRTEM). Excluding the larger microstructures (*vide infra*), the average size of the particles from SEM images is found to be around −100 nm. DLS measurements verify the size of the particles for the same sample. The sample is serially diluted three orders of magnitude for these measurements. Particles were measured in triplicate. Figure 1D presents the size distribution by intensity obtained from DLS analysis. It can be noticed that there is some general agreement with only a small difference (−10–20%) between the individual size of the particles from SEM on the one hand and that determined by DLS measurements on the other hand. There are some large microstructures that are noticed in the SEM image, which can be attributed to salt precipitation during the drying process. The general qualitative agreement with a small 10–20% difference between electron microscopy and light scattering particle size determination is also consistent with previous work from our group and others [19,21,22]. Multiple factors explain such small size differences, including variation in drying vs hydration conditions, which can lead to swelling, de-swelling/contraction, salt precipitation, and/or agglomeration vs monodispersion [23,24].

A standard ninhydrin assay protocol measured the amine content (Sigma). A calibration curve was acquired using a Lambda 900 UV-Vis-NIR spectrometer (PerkinElmer, Waltham, MA, USA) that was equipped with Extrasil Quartz cuvettes. Equal volumes of ninhydrin (1.00 mL) were added to five vials, and AMA (50 µM) was added in increasing volumes to each vial, from 0 to 2.00 mL, to create a linear range of concentration. The samples were heated at 100 °C for 10 min., and after cooling to ambient temperature (22 °C to 27 °C), the nanoparticles’ absorbance was measured by UV-Vis spectroscopy. Absorbances were recorded at 570 nm. The amine-bearing particles were then added to another vial containing the same volume of ninhydrin that was used in the determination of the amine content and heated for 10 min. at 100 °C, and the vial containing the sample was immersed in an ultrasound bath for one minute to suspend the particles. Amine monomer conversion was determined to be 81.3% by ninhydrin assay.

## 3. Results and Discussion

The synthesized particles had a total average dispersity of PDI = 0.048, which is well below Malvern Panalytical’s minimum acceptable PDI of 0.7.

The low dispersity is beneficial for biological studies, where size dispersity affects the cytotoxicity of nanoparticle therapies [25,26,27].

**Figure 2:**
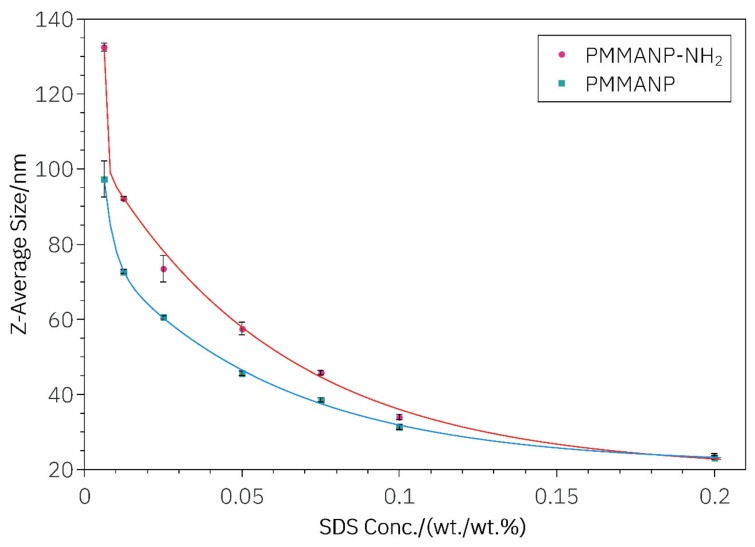
Size tunability of PMMANPs and H_2_N-PMMANPs.

As shown in Table 1 and Table 2 as well as in Figure 2, the change in size of both types of particles with respect to SDS concentration followed a second order exponential decay relationship, which was fitted using the scaled Levenberg–Marquardt algorithm, given by the general equation:

(1) $$A_{1}e^{\left(-\frac{x}{t_{1}}\right)}+A_{2}e^{\left(-\frac{x}{t_{2}}\right)}+y_{0}$$

In Table 3, *A*_1_ and *A*_2_ are the first and second decay constants, respectively; *t*_1_ and *t*_2_ are the first and second decay times, respectively; and, y_0_ is the y-intercept.

(2) $$\mathrm{Size}\;\mathrm{PMMANP}\;(\mathrm{nm})=\left[\left(1.66\times 10^{2}\right)\left(nm\right)\times e^{\left(-\frac{x\;\left(\frac{wt}{wt}\%\right)}{3.07\times 10^{-3}\;\left(\frac{wt}{wt}\%\right)}\right)}\right]+\left[\left(6.04\times 10^{12}\right)\left(nm\right)\times e^{\left(-\frac{x\;\left(\frac{wt}{wt}\%\right)}{5.68\times 10^{-2}\;\left(\frac{wt}{wt}\%\right)}\right)}\right]+21.43\left(nm\right)$$

(3) $$\mathrm{Size}\;H_{2}N-\mathrm{PMMANP}\;(\mathrm{nm})=\left[\left(8.78\times 10^{6}\right)\left(nm\right)\times e^{\left(-\frac{x\;\left(\frac{wt}{wt}\%\right)}{6.12\times 10^{-4}\;\left(\frac{wt}{wt}\%\right)}\right)}\right]+\left[\left(8.96\times 10^{1}\right)\left(nm\right)\times e^{\left(-\frac{x\;\left(\frac{wt}{wt}\%\right)}{5.82\times 10^{-2}\;\left(\frac{wt}{wt}\%\right)}\right)}\right]+19.93\left(nm\right)$$

A rigorous mathematical investigation is not performed since the goal of this study was to develop a practical relationship between particle size and surfactant concentration. The constants *A*_1_ and *A*_2_ may correspond to the absolute concentrations of monomers, or ratios of monomer to surfactant. The t_1_ and *t*_2_ decay constants may represent diffusion coefficients between the organic and aqueous fractions of the reaction emulsion. This explanation is consistent with the observed results, because the surfactant concentration modulates the diffusion rate of reactants and growing polymers between organic and aqueous phases. Consequently, the equilibrium of Ostwald ripening will be modulated [28]. In order to verify that the equation relating particle size and SDS concentration can determine particle size as a function of SDS concentration, an SDS concentration between the lowest and highest concentrations of SDS tested was selected and PMMANPs and H_2_N-PMMANPs were made according to our already detailed modified method of Yuan et al. PMMANPs and H_2_N-PMMANPs had Z-average sizes of 38.57 nm and 45.76 nm, respectively, when compared to their formula-calculated values of 36.65 nm and 44.67 nm. This is a difference of 1.92 nm and 1.09 nm, respectively. Overall, PMMANPs and H_2_N-PMMANPs had total average differences of 2.74 nm and 4.32 nm, respectively, from their equation-calculated values. These values are within the instrumental error of the Malvern Zetasizer, which was used to measure the Z-average size of the particles. While others have investigated size control and elucidated relationships between size and SDS concentration, there have been no studies in the size range tested herein, which describe a practical relationship that can be used for the mathematical determination of the exact reagent amounts that are necessary for making a desired size of PMMANP or H_2_N-PMMANP. Many investigations have shown a relationship between particles in the size range of 300 to 1000 nm, as well as 1–20 nm, but few have explored the range occupied by these H_2_N-PMMANP and PMMANP particles [29]. The size range investigated in the present study is interesting from a nanomaterials synthesis standpoint, because there is a nonlinear response of particle size to surfactant concentration, which is consistent with the number of reactants participating in nanoparticle formation. This contrasts with other studies, which report a linear response of particle size to surfactant concentration. The second-order exponential decay, as opposed to a linear or single exponential decay relationship, is likely attributed to multiple reactants, which affect size outcome in the particular size range of the particles in this study.

## Figures and Tables

**Table 1:** Actual *vs.* Formula-calculated Sizes of poly(methyl methacrylate) (PMMA) nanoparticles (PMMANPs).

SDS (wt./wt.%)	PMMANP Z-Ave (nm)		PDI
	Actual	Calculated	
0.200	23.19 ± 1.57	24.97	0.09
0.100	31.31 ± 1.64	31.88	0.06
0.075	38.57 ± 2.74	37.44	0.03
0.050	45.61 ± 2.73	46.29	0.05
0.025	60.49 ± 2.93	60.37	0.04
0.013	77.70 ± 2.95	72.47	0.04
0.008	97.36 ± 11.77	91.74	0.03

**Table 2:** Actual *vs.* Formula-calculated Sizes of H_2_N-PMMANPs.

SDS (wt./wt.%)	H_2_N-PMMANP Z-Ave (nm)		PDI
	Actual	Calculated	
0.200	23.40 ± 1.53	24.68	0.08
0.100	34.00 ± 1.60	35.06	0.06
0.075	45.76 ± 2.81	42.46	0.03
0.050	57.50 ± 4.67	54.53	0.05
0.025	73.56 ± 6.35	74.28	0.04
0.013	92.22 ± 2.97	95.03	0.04
0.008	132.53 ± 3.23	125.22	0.03

**Table 3:** Respective Values for Constants in the Fitted Function.

Constant	PMMANP	H_2_N-PMMANP
A_1_ (nm)	1.67 × 10^2^	8.78 × 10^6^
A_2_ (nm)	6.04 × 10	8.96 × 10
t_1_ (wt./wt.%)	3.07 × 10^−3^	6.12 × 10^−4^
t_2_ (wt./wt.%)	5.68 × 10^−2^	5.82 × 10^−2^
y_0_ (nm)	21.43	19.93
